# Fall prevention interventions for older community-dwelling adults: systematic reviews on benefits, harms, and patient values and preferences

**DOI:** 10.1186/s13643-020-01572-7

**Published:** 2021-01-09

**Authors:** Jennifer Pillay, John J. Riva, Laure A. Tessier, Heather Colquhoun, Eddy Lang, Ainsley E. Moore, Brett D. Thombs, Brenda J. Wilson, Amanda Tzenov, Catherine Donnelly, Marcel Émond, Jayna Holroyd-Leduc, Jamie Milligan, Diana Keto-Lambert, Sholeh Rahman, Ben Vandermeer, Andrea C. Tricco, Sharon E. Straus, Sonia M. Thomas, Bradley R. Mitchelmore, Elizabeth Rolland-Harris, Lisa Hartling

**Affiliations:** 1 Alberta Research Centre for Health Evidence, University of Alberta, 11405 87 Avenue, Edmonton, AB T6G 1C9 Canada; 2 Department of Family Medicine, McMaster University, Hamilton, Canada; 3 Global Health and Guidelines Division, Public Health Agency of Canada, Ottawa, Canada; 4 Occupational Science and Occupational Therapy Department, University of Toronto, Toronto, Canada; 5 Cumming School of Medicine, University of Calgary and Alberta Health Services, Calgary, Canada; 6 Faculty of Medicine, McGill University, Montreal, Canada; 7 Community Health and Humanities, Faculty of Medicine, Memorial University of Newfoundland, St. John’s, Canada; 8 Memorial University of Newfoundland, St. John’s, Canada; 9 School of Rehabilitation Therapy, Queen’s University, Kingston, Canada; 10 Department of Family Medicine and Emergency Medicine, Université Laval, Quebec City, Canada; 11 Department of Medicine, University of Calgary, Calgary, Canada; 12 Schlegel Specialist in Mobility and Falls, Schlegel-UW Research Institute for Aging, Department of Family Medicine, McMaster University, Hamilton, Canada; 13 Knowledge Translation Program, Li Ka Shing Knowledge Institute, St Michael’s Hospital, Unity Health Toronto, Toronto, Canada

**Keywords:** Fall prevention, Systematic review, Patient values and preferences, Guideline, CINeMA, GRADE

## Abstract

**Background:**

An estimated 20–30% of community-dwelling Canadian adults aged 65 years or older experience one or more falls each year. Fall-related injuries are a leading cause of hospitalization and can lead to functional independence. Many fall prevention interventions, often based on modifiable risk factors, have been studied. Apart from the magnitude of the benefits and harms from different interventions, the preferences of older adults for different interventions as well as the relative importance they place on the different potential outcomes may influence recommendations by guideline panels. These reviews on benefits and harms of interventions, and on patient values and preferences, will inform the Canadian Task Force on Preventive Health Care to develop recommendations on fall prevention for primary care providers.

**Methods:**

To review the benefits and harms of fall prevention interventions, we will update a previous systematic review of randomized controlled trials with adaptations to modify the classification of interventions and narrow the scope to community-dwelling older adults and primary-care relevant interventions. Four databases (MEDLINE, Embase, Cochrane Central Register of Controlled Trials, Ageline), reference lists, trial registries, and relevant websites will be searched, using limits for randomized trials and date (2016 onwards). We will classify interventions according to the Prevention of Falls Network Europe (ProFANE) Group’s taxonomy. Outcomes include fallers, falls, injurious falls, fractures, hip fractures, institutionalization, health-related quality of life, functional status, and intervention-related adverse effects. For studies not included in the previous review, screening, study selection, data extraction on outcomes, and risk of bias assessments will be independently undertaken by two reviewers with consensus used for final decisions. Where quantitative analysis is suitable, network or pairwise meta-analysis will be conducted using a frequentist approach in Stata. Assessment of the transitivity and coherence of the network meta-analyses will be undertaken. For the reviews on patient preferences and outcome valuation (relative importance of outcomes), we will perform de novo reviews with searches in three databases (MEDLINE, PsycInfo, and CINAHL) and reference lists for cross-sectional, longitudinal quantitative, or qualitative studies published from 2000. Selection, data extraction, and risk of bias assessments suitable for each study design will be performed in duplicate. The analysis will be guided by a narrative synthesis approach, which may include meta-analysis for health-state utilities. We will use the CINeMa approach to a rate the certainty of the evidence for outcomes on intervention effects analyzed using network meta-analysis and the GRADE approach for all other outcomes.

**Discussion:**

We will describe the flow of literature and characteristics of all studies and present results of all analyses and summary of finding tables. We will compare our findings to others and discuss the limitations of the reviews and the available literature.

**Systematic review registration:**

This protocol has not been registered.

**Supplementary Information:**

The online version contains supplementary material available at 10.1186/s13643-020-01572-7.

## Background

### Burden of falls

An estimated 20–30% of older Canadian adults (> 65 years old) living in the community experience one or more falls each year [1]. In older adults, fall-related injuries (limiting normal activities) occur more frequently in females than in males (e.g., 67 vs. 46 per 1000 in 2010) and increase with age—rates for those ≥ 85 years of age are about double for those 65 to 74 years of age [1].

Approximately 10% of falls among older adults result in a fracture [2, 3]. The majority of cost, morbidity, and mortality generated by fall-related injuries are attributed to peripheral fractures, especially at the hip, rather than vertebral fractures or injuries to the soft tissues or organs [1, 4]. Less serious injuries, such as bruising, lacerations, and sprains, can still lead to pain, reduced function, and substantial costs to the individual and healthcare system [5]. In Canada, the leading causes of fall-related injuries in older adults were walking (16% on snow or ice and 45% on other surfaces) and accidents going up or downstairs (13% of injuries) [1]. The 2009/10 Canadian Community Health Survey found that the most common fall-related injury in older adults was a fracture (35%) followed by sprains/strains (30%) [1]. Emergency room visits following a fall are not limited to those experiencing a fracture; the survey also found that of older adults who reported seeking treatment related to a fall, most (67%) went to an emergency room. Injuries resulting from a fall are a leading cause of hospitalizations among Canadian adults aged > 65 years, and the length of hospitalization from fall-related injuries is on average 9 days longer (e.g., 21 vs. 12 days) than for all causes of hospitalization [1]. Based on data from Canadian acute care hospitals, 17% of older adults who were hospitalized for a fall were moved from living in the community to a continuing care facility [6]. Many older people also experience psychological difficulties after falls, including a fear of falling or loss of confidence, which may contribute to further falling [7]. Falls have been associated with reduced quality of life [8] and can lead to loss of function in activities of daily living (ADLs), thereby impacting independence and overall health outcomes [9].

### Risk factors and screening for risk

Falls often are a result of a complex combination of risk factors, interacting to cause an inability to maintain or regain one’s balance [1]. Risk factors may be classified as (i) biological, related to disease(s), and the natural aging process (e.g., balance and gait deficiency, acute or chronic health conditions, cognitive impairment, low vision); (ii) behavioral, such as the use of unsuitable/poorly maintained assistive devices including footwear and clothing, fear of falling, the use of certain medications (e.g., psychotropic, sedatives, hypnotics), and vitamin D intake to improve the function of the skeletal muscle [10, 11]); (iii) social and economic (e.g., social isolation, poverty, poor access to healthcare); and (iv) environmental from factors in the community (e.g., building entrances, lack of handrails), living environment (e.g., type of furniture, home clutter), and/or related to weather and climate (e.g., icy surfaces) [1].

Screening patients for their risk for falls has been considered, with the aim of determining who may benefit the most from further assessment and/or an intervention to prevent falls. Many different methods for screening have been developed or considered, relying on single or multiple-item history questions, self-report measures/questionnaires (e.g., Falls Efficacy Scale [12]), or performance-based measures (e.g., 30 Second Chair Stand [13], Berg Balance Scale [14], and Timed Up and Go [15]). However, no single screening method has demonstrated to be simple and highly accurate for predicting risk, intervention effects may not be modified by fall risk status, and we are not aware of any trials or systematic reviews on screening effectiveness. A 2018 systematic review of 26 assessment tools for falls risk in older adults found that no single tool showed sufficiently high predictive validity (e.g., sensitivities and area-under-the curves ranged from 63 to 76% and 0.76 to 0.81, respectively, for those used in community-dwelling populations) to differentiate between people at high versus low risk of falls [16]. The authors concluded that clinicians should consider using at least two assessment tools together to better evaluate their patients’ risk. Likewise, when evaluating the predictive ability of single and combined use of medical history questions, authors of another review concluded that no single question emerged as a powerful predictive tool, but that querying several factors together (i.e., fall history, difficulty with ADLs, the use of an ambulatory device, concern about falling, and use of psychoactive medication) could be useful (e.g., using a calculation of cumulative post-test probability to indicate that a patient’s risk for falling would change from 30% without screening to ≥ 60%) [17]. The time burden to adopt a multiple tool-based or question strategy may lead to unacceptably low uptake by primary care providers due to competing demands that occur in the care of older patients, often with multiple comorbidities to manage during a clinical encounter. Further, several reviews of fall prevention intervention studies have found no significant difference in effects for exercise, multiple component, or multifactorial interventions based on whether or not above-average or high-fall risk was used for study inclusion [18–20].

### Fall prevention interventions

Many interventions for preventing falls have been studied. These are often based on known, modifiable risk factors for falling. Most fall prevention interventions can be classified according to the internationally accepted taxonomy developed by the Prevention of Falls Network Europe (ProFANE) Group [21]. A major feature of the taxonomy is the distinction between different categories and combinations of interventions. Interventions may comprise *single-component interventions*, involving one or a combination of two or more interventions (e.g., gait and balance training with strength/resistance exercises) from the same category (e.g., exercise), or interventions where more than one intervention from different categories are offered to everyone (*multiple component interventions*) or tailored based on an individual’s risk assessment (*multifactorial interventions*). Single and multiple component interventions may include some form of fall risk assessment or be directed towards those with one or more particular risk factor(s), but they do not tailor the intervention components to each individual’s risk as do multifactorial interventions. Some of the interventions within the taxonomy are more applicable than others to the general population of community-dwelling older adults and the practice of primary care (i.e., first-contact, accessible, continued, comprehensive and coordinated care [22]). These may be provided directly by primary care providers (e.g., vitamin D supplementation, nurse-led education on falls risk and prevention), by an inter-professional team of providers (e.g., exercise and cognitive-behavioral therapy), through referral to an allied health care provider (e.g., environmental/home hazard assessment), or in the community (e.g., patient-initiated attendance at Tai Chi classes). Other interventions, such as management of urinary incontinence or cataract surgery, target populations having a specific diagnosis or condition and, when given alone, are not considered to address the primary aim of fall prevention. Interventions may differ depending on the population (e.g., general vs. increased risk based on recruitment from emergency departments vs. frail) and delivery setting (e.g., community vs. nursing homes vs. hospitals). They may also focus on the primary prevention of a fall or secondary prevention of subsequent falls. Some interventions may be better considered “add-on” strategies, that are likely insufficient on their own to prevent falls, but rather used to enhance the uptake or implementation of a main intervention (e.g., patient appointment reminders, clinician training in exercise therapy, or fall risk assessment).

### Relevance of values and preferences

Health-care decision-making is influenced by the health effects of interventions as well as people’s values and preferences [23]. While acceptance rates across various types of fall prevention interventions, on average, appear quite high (e.g., approximately 70% of older adults agree to participate in studies regardless of eligibility [24]), there is evidence on stated preferences for different types and/or formats of interventions (e.g., [25–27]) that could inform decisions about which interventions to recommend in general and to specific populations. Moreover, preferences for or against an intervention are viewed as a consequence of the relative importance people place on the expected or experienced health outcomes it incurs [28]. When considering multiple different types of interventions where the impact on different outcomes may vary between types, the relative importance placed by patients on the different potential outcomes may influence which interventions are considered more or less effective.

### Aims and rationale for reviews

The findings of three systematic reviews will be used by the Canadian Task Force on Preventive Health Care—supplemented by input from patient and organizational stakeholder consultations and by other sources of information on feasibility, acceptability, costs/resources, and equity―to make recommendations for primary care providers on fall prevention interventions. The following key questions (KQs) will be answered.

- KQ1: What are the benefits and harms of interventions compared with usual care to prevent falls in community-dwelling adults aged 65 and older?
- KQ2: How do community-dwelling adults aged 65 and older weigh the potential benefits and harms of interventions to prevent falls?
- KQ3: What are the preferences of community-dwelling adults aged 65 and older regarding different interventions demonstrated to prevent falls?

A comprehensive search for systematic reviews related to our KQs published between 2014 and 2019 found that while many systematic reviews (> 80) exist, most have focused on specific types of interventions (e.g., exercise) and/or specific populations (e.g., cognitively impaired). Although some recent reviews could be considered as closely meeting our aim for KQ1 on benefits and harms of various interventions [18, 29–31], no review matches this review’s scope fully in terms of their population, interventions, comparators, outcomes, timing, and setting (i.e., PICOTS). To avoid duplication of effort and build on others’ work, we will rely on a review with the broadest scope/PICOTS [29] for identification and data extraction of studies related to KQ1 on benefits and harms. We will update the literature base and make adaptations where suitable to meet the narrower scope determined by the Task Force (e.g., exclude interventions delivered exclusively in hospital or nursing home settings). We did not identify an existing review fully answering KQ2 on the relative importance of outcomes or KQ3 on preferences for different interventions, respectively, and will therefore conduct de novo reviews for these two questions.

## Methods

The Evidence Review and Synthesis Centre at the University of Alberta’s Alberta Research Centre for Health Evidence will complete the reviews (JP, DK-L, BV, SR, LH). The reviews will be developed, conducted, and prepared according to the Task Force methods [32], using methods guided by the Cochrane Handbook [33] and Grading of Recommendations Assessment, Development and Evaluation (GRADE) working group [23, 34, 35]. This protocol follows reporting standards [36]. The review for KQ1 on the benefits and harms of fall prevention interventions will be conducted in collaboration with the authors of the review which is being adapted (ACT, SES, SMT) [29]. The protocol was reviewed by peer–reviewers and organizational stakeholders (*n* = 9). This final version of the protocol has been approved by the entire Task Force.

A working group of Task Force members (JJR, HC, EL, AEM, BDT, BJW, AT) and content experts (CD, ME, JH-L, JM) was formed for development of KQs and PICOTS. Task Force members chose and rated outcomes in terms of their importance for creating a recommendation, according to methods of GRADE [35]. Outcome ratings were finalized after input from an outcome rating exercise and focus groups conducted with a sample of older adults in Canada, by an independent group, led by SES, with expertise in knowledge translation from St. Michael’s Hospital in Toronto, Ontario. Eight outcomes were considered critical for decision making (i.e., rated 7 or above on a scale of 1–9) by the Task Force: number of fallers, number of falls, number of injurious falls, number of fractures, number of hip fractures, residential status/institutionalization, health-related quality of life, and functional status. Intervention-related adverse effects (AEs; any or serious) were rated as important (i.e., rated 4–6) and included.

The Science Team of the Global Health and Guidelines Division at the Public Health Agency of Canada (PHAC) (LAT, BM, ERH) provided assistance and input on Task Force methodological considerations during the development of the protocol.

### Eligibility criteria

Tables 1, 3, and 4 outline each KQ’s study eligibility criteria (i.e., PICOTS). Table 2 is specific to the components of fall prevention interventions, and study comparators, of interest.

**Table 1:** Eligibility criteria for key question 1

	Inclusion criteria	Exclusion criteria
**Population**	Adults living in the community, aged 65 or older. Community living consists of living at home or in independent living/retirement facilities where no or minimal assistance (e.g., help with one activity of daily living [ADL; e.g. bathing] or Instrumental ADL, e.g. cooking) is provided Studies recruiting adults living in the community under the age of 65 may be included if ≥ 80% of the participants are aged 65 or older. If the proportion of the participants aged 65 or older is not available, studies may be included if the participants’ mean age minus one standard deviation is equal to or greater than 65.	Studies with recruitment based exclusively on one or more specific diagnoses. Excluded populations include, but are not limited to: • Stroke • Parkinson’s (neurodegenerative conditions) • Severe dementia (will include if all mild-to-moderate cognitive impairment) • Long-term care facilities • Housebound • Severe frailty (with protocol for addressing falls or for falls risk assessment in place) • Impaired balance (severe) • Community-dwelling and receiving long-term, intensive nursing care • Visual impairment (severe) • Hospitalized patients • Acute fracture • Confirmed vitamin D deficiency
**Interventions**	Single component, multiple component or multifactorial interventions in which the primary objective is to prevent falls. Intervention components that can be classified using the ProFANE Taxonomy (Table 2).	Interventions that cannot feasibly or readily be delivered or referred to by a wide variety of primary care providers (see exclusions in Table 2). Interventions that are not directly focused on the cascade of falls (i.e., falls prevention must be primary aim of intervention). Single interventions that are exclusively screening/assessment tools and/or quality improvement (“add-on”) strategies.
**Comparator**	• Usual care (e.g., no additional care focusing on falls; may include wait-list, attention control, pamphlet or generic health education, placebo) • Non- or minimally active intervention (e.g., brief pamphlet on falls risk, social visits) • Another intervention to prevent falls; for critical outcomes where NMA is conducted only, and if classified differently according to ProFANE taxonomy and our coding	
**Outcomes**	**Critical** • Falls (i.e., total number of falls per unit of person-time) • Fallers (i.e., number of people falling one or more times during follow-up) • Injurious falls (one used per study using a hierarchy: falls leading to hospitalization, falls requiring emergency department visit, falls requiring physician visit, any injurious fall); preferentially the number of people with one or more injurious falls, but will include total number if necessary • Fractures (only fall-related, if reported; preferentially the number of people with one or more fractures, but will include total number if necessary) • Hip fractures (only fall-related, if reported) • Residential status/institutionalization (number of people newly admitted to residential care) • Health-related quality of life (validated scales, e.g., SF-12 and/or SF-36 Physical and Mental Components, EQ 5D VAS, EuroQol EQ-5D) • Functional status: (i) validated scales including activities of daily living and instrumental ADLs [composite scores only], (ii) number of people with new/increased need for homecare assistance; (iii) other validated scales will be considered **Important** • Intervention-related adverse effects (AEs) as defined by study (people experiencing one or more AEs; individual serious AEs)	
**Timing**	Follow-up duration: ≥ 3 months after randomization	
**Delivery Setting**	Any relevant to primary care (primary care, community [home or other]). Interventions can be initiated in the emergency department, but cannot be entirely delivered in the emergency department.	Settings not relevant to primary care and targeting general community-dwelling population (e.g., workplaces, inpatient settings, specialist settings, interventions entirely delivered in the emergency departments, nursing/long-term care homes).
**Study design**	Randomized controlled trials (all designs including parallel, cluster, crossover, multifactorial)	• Editorials • Commentaries • Studies only published/available as conference proceedings, letters, or other gray literature (e.g., government reports), unless information on study design (e.g., eligibility criteria, participant characteristics, intervention characteristics) is described sufficiently and results are confirmed as final (accessible online or via author contact)
**Language of full text**	English or French	
**Dates of publication**	Any

**Table 2:** Modified ProFANE taxonomy [21] of interventions for inclusion and description of add-on strategies and comparators

**Category of intervention**^**a**^	**Single interventions within category**^**b**^
Exercise	Gait, balance, coordination, and functional training; strength/resistance exercises; flexibility exercises; 3D training (e.g., Tai Chi, dance, yoga); general physical activity; endurance training; others (e.g., exergame, aquatic); mixed exercises (i.e., 2 or more exercise components)
Medication (drug provision)	Vitamin D (+/−calcium) supplementation; sunlight interventions; excluding calcium alone, anti-osteoporosis medications or others used for specific conditions where fall prevention is secondary aim (e.g., diabetes medication, urinary antispasmodics)
Medication (review & modification)	Medication withdrawal, dose reduction or increase, substitution (may be delivered directly to patient or focus on provider education)
Nutrition therapy	Dietary counselling; excluding single dietary supplements and fluid therapy
Psychological	Cognitive (behavioral) interventions
Environment/Assistive Technology (furnishings and adaptations to homes and other premises/direct action)	Relocation, entrances, flooring, lighting, installation of grab bars in bathrooms, handrails for stairs, others
Environment/Assistive Technology (aids for personal mobility and protection)	Walking aids, clothes, orthotics, or anti-slip devices for shoes; excluding protective aids to reduce fractures from falls (e.g., hip protectors) and comprehensive podiatry assessment
Environment/Assistive Technology (aids for communication and signaling)	Alarm systems to prevent falls; excluding alarms to signal a fall, hearing aids, or optical aids unless part of a multiple component intervention
Environment/Assistive Technology (aids for communication and signaling)	Vision assessment and treatment
Knowledge/education interventions	Written material, videos, and lectures about reducing falls; excluding pamphlets
**Category of intervention add-on strategy**^**b**^	**Interventions within category**
Social environment (clinic quality improvement)	Staff ratio, staff training, service model change, clinician reminders, audit and feedback, case management, referral (not for falls risk assessment or interventions which is captured by delivery variable); training and education to deliver the main interventions will not be counted (e.g., training of staff to deliver CBT or medication review)
Social environment (patient quality improvement)	Telephone support or reminders about appointments or aspects of care, caregiver training, homecare services, promotion of self-management (e.g., goal setting, action planning)
**Control groups**^**c**^	**Examples**
Usual care (UC)	May include wait-list control, placebo (for vitamin D interventions), or session or pamphlet on general health or active living; excluding studies where UC involves assessments (e.g., comprehensive geriatric) or interventions (potentially reducing falls) that are provided to all participants and not considered UC for the general community-dwelling population ≥ 65 years of age^d^
Non- or minimally active intervention (information)	UC as well as basic assessment related to falls risk factors without follow-up; brief pamphlet on falls risk or session on gentle exercises
Non- or minimally active intervention (social engagement)	UC as well as social visits/engagement, including group sessions

Abbreviations: *CBT* cognitive behavioral therapy, *UC* usual care

^a^Interventions will also be categorized as single-component (e.g., one or more single interventions from a single category), multiple component (more than one single intervention from different categories offered to all people), and multifactorial (one or more single interventions are offered from different categories, based on an individual risk assessment). Multifactorial interventions will be assumed to include an assessment involving multiple falls risk factors, whereas other interventions will also be assumed to include some form of assessment (e.g., gait, medication review, dietary assessment)

^b^Excluded interventions and add-on quality improvement strategies cannot be the only intervention provided, but may be an included component of a multiple component or multifactorial intervention. Single interventions not specified in the table will be considered

^c^All three possible control groups will be considered for the analysis; the forest plots will then be visually inspected to determine the degree of similarity between theses nodes, and they may be joined into 1 or 2 nodes thereafter

^d^We will seek clinical input from the WG and clinical/topic experts before excluding any studies that may have UC that is not relevant/generalizable to the target population

The main population of interest for all KQs is adults aged 65 or older living in the community, that is, at home or in independent living/retirement facilities where no or minimal assistance is provided. We will include studies only recruiting people who have never fallen as well as those that include people who have a history of falls. For KQ2, when looking at the valuation/importance of the outcomes of fractures and transfer to residential status/institutionalization, we will also include studies of populations newly admitted to residential care/nursing homes. We will exclude studies with recruitment based exclusively on one or more specific medical diagnoses (e.g., stroke, Parkinson’s disease), because these populations are expected to require fall prevention interventions and management/usual care that are substantially different from those applicable to the general population of community-dwelling adults. For KQ2 and 3, studies may include family members or caregivers who participate on behalf of people with cognitive impairment or otherwise unable to understand the study procedures.

For KQ1 on benefits and harms, we will include studies with at least one eligible single intervention as described in Table 2, of those chosen by the Task Force working group to reflect interventions having a primary aim to prevent falls in a broad population of community-dwelling older adults, and delivered in, or referable from, a primary care setting. We will exclude interventions that are solely used for screening or assessment, or as “add ons” to improve the uptake or implementation of interventions targeted at preventing falls but not proposed to reduce falls themselves. Participants can be recruited in hospitals, but the intervention must be primarily delivered outpatient in primary care or the community.

The main KQ1 comparator is usual care (UC), which is considered the medical and health care received by the target population within primary care that does not include any specific intervention to reduce falls. We will also include studies with a control having a non/minimally active intervention such as a pamphlet on falls risk or social engagement activities. We will seek clinical input in cases where there is uncertainty about whether the UC (as described by authors) is applicable to the general population of interest; if not applicable (e.g., comprehensive geriatric assessment is provided to all patients), the study will be excluded. Although the main interest of the Task Force is the effects of interventions versus UC, rather than the relative effects between different types of interventions, for critical outcomes, we will include head-to-head trials of different interventions and conduct network meta-analysis (NMA) to maximize the amount of data used and to generate estimates of the effects versus UC for those interventions that have not been (or have been minimally) studied in direct comparison with UC [37–39]. Studies that only compare different interventions that are both defined within one single intervention of our taxonomy (Table 2; e.g., different doses of vitamin D or intensities of strength training) will be excluded. Final inclusion of head-to-head comparisons will be based on the intervention (node) configurations in the final NMAs (see Data Synthesis for Key Question 1). For outcomes for which we do not undertake NMA, we will define the interventions as per the nodes used in the NMAs and only include studies using comparisons with UC or non-/minimally active interventions.

We will include randomized controlled trials (RCTs), of any design, with at least 3 months of follow-up after randomization to adequately capture the potential effects on the outcomes. Apart from English language reports, we will include those reported in French, as the Task Force considers reports published in both official languages in Canada (English and French). Literature suggests that language restrictions in systematic reviews on conventional medicine topics do not appear to bias results from meta-analyses [40, 41]. No restrictions will be placed on publication status, date, country, or risk of bias.

For KQs 2 and 3, the effects of the interventions are not of interest but rather the valuation/relative importance of the critical outcomes (KQ2) and the preferences of older adults for different interventions or intervention attributes (KQ3). The eligibility criteria for the studies in KQ2 align with those described by the GRADE working group [23, 28, 34]. For KQ2, we will prefer studies comparing two or more of the relevant outcomes (e.g., falls versus fractures) and/or with a comparison with a healthy population; studies without these will be considered if evidence is lacking on the importance of one or more outcomes. KQ3 will be conducted after KQ1, because we will only examine the preferences between different interventions, or between different attributes of interventions, that are shown to be effective by the KQ1 analysis. The attributes of interest will also be decided after completion of KQ1, but prior to study selection for KQ3. We will use a hierarchy of study designs for KQs 2 and 3, in order to prioritize the most informative study designs for each KQ (see Tables 3 and 4). Qualitative studies would be very informative if the KQs were exploring reasoning (e.g., beliefs, barriers, expectations) behind the preferences, but the most relevant evidence on preferences as specified for these KQs is quantitative in nature. Studies reported in English or French will be sought. No restrictions will be placed on publication status, country, or risk of bias. We will limit inclusion to studies published on or after 2000 because it is expected that people’s preferences change over time and because we expect a large proportion (> 90%) of studies on fall prevention interventions to be conducted after this date [29].

**Table 3:** Eligibility criteria for key question 2

Criteria	Inclusion	Exclusion
Population	Adults living in the community, aged 65 or older; when looking at importance of outcome of residential status, population may be awaiting or newly admitted to residential care Studies recruiting adults < 65 years will be included if ≥80% of the participants are aged 65 or older, if participants’ mean age minus one standard deviation is ≥ 65 years, or if results are provided for those ≥ 65 years. Family members or caregivers may serve as participants on behalf of an older adult with cognitive impairment or otherwise unable to understand the study procedures.	Studies with recruitment based exclusively on one or more specific diagnoses. Excluded populations include, but are not limited to: • Stroke • Parkinson’s (neurodegenerative conditions) • Severe dementia • Long-term care facilities (unless newly admitted) • Housebound • Severe frailty (with protocol for addressing falls or for falls risk assessment in place) • Impaired balance (severe) • Community-dwelling and receiving long-term, intensive nursing care (unless newly acquired need) • Visual impairment (severe) • Hospitalized patients (unless with acute fracture or injury from fall) • Confirmed vitamin D deficiency
Exposure(s)	• Experience with critical outcome(s) of interest, or • Exposure to clinical scenario(s) or information about potential critical outcome(s) and/or estimate(s) of effect on outcomes from falls prevention interventions, or • No experience or exposure to information about critical outcomes, but authors are soliciting probability trade-offs (e.g., number of adverse events from interventions to make one fewer fall worthwhile) or ratings of different potential critical outcomes Focus of study is on consideration of possible, or assessment of experienced, outcomes related to falls prevention that are considered critical by the Canadian Task Force on Preventive Health Care (see outcomes Table 1). For fractures, the main three “sub-outcomes” considered for this KQ will be “any fracture attributed to a fall”, “any fracture” and “a single hip fracture”.	
Comparison(s)	a) Experience or exposure to scenarios or information about a different critical outcome (e.g., falls vs. any fracture, hip vs. “any fracture”) b) Healthy state without critical outcome (for utility studies only) c) No comparison (for utility studies only, if information from comparisons a or b are not available for a particular outcome)	
Outcomes	a) Utility values/weights for the potential outcomes/health states b) Non-utility, quantitative information about relative importance of different outcomes, e.g., rating scales using ordinal or interval variables, ranking; preference for or against interventions [attendance, intentions, or acceptance] or preferred type of intervention based on different outcome risk descriptions, strength of associations between outcome ratings and behaviors or intentions for falls prevention interventions c) Qualitative information indicating relative importance between outcomes Data must relate to the outcomes considered critical to the Task Force (Table 1); for studies measuring the health state utility of a fracture or those residing in residential homes/facilities, attribution to a fall will be prioritized, as possible Outcome groupings (a) to (c) above will be included in a hierarchical manner	
Timing	Follow-up duration: any or none	
Setting	Any	
Study Design and Publication Status	Any cross-sectional or longitudinal quantitative or qualitative study design using the methods described below: Methods: a) Utility values/weights for health states measured directly using time trade-off*, standard gamble**, visual analogue scales, conjoint analysis with choice experiments or probability trade-offs b) Utility values/weights measured or estimated indirectly, e.g., a person’s health status is elicited along several dimensions using a questionnaire (e.g., EuroQol-5D), then a preference for that particular health state is derived, based on values obtained from previous populations c) Surveys or questionnaires with questions providing non-utility, quantitative information about relative importance of different outcomes; may be investigating decision aids d) Qualitative studies providing information indicating relative importance between benefits and harms Study design groupings (c) and (d) will be included only if insufficient data is available from (a) and (b)	• Commentaries, opinion, editorials, case reports, and reviews • Studies only published/available as conference proceedings or other grey literature (e.g., government reports), unless information on study design (e.g., eligibility criteria, participant characteristics, presentation of scenarios) is available (accessible online or via author contact) and sufficient to assess methodological quality.
Language	English or French	
Publication date	2000-present	

*Time trade-off measures the value placed on attributes of a commodity by requiring individuals to choose between different scenarios, where in each scenario the commodity in question has varying levels of different attributes

**Standard gamble approaches require that respondents choose between a lifetime in a certain health state or a gamble between different health states, whereas time trade-off requires respondents to choose between living for a period in less than perfect health, as opposed to a shorter period in perfect health

**Table 4:** Eligibility criteria for key question 3

Criteria	Inclusion	Exclusion
Population	Adults living in the community, aged 65 or older Studies recruiting adults < 65 years will be included if ≥ 80% of the participants are aged 65 or older, if participants’ mean age minus one standard deviation is ≥ 65 years, or if results are provided for those ≥ 65 years. Family members or caregivers may serve as participants on behalf of an older adult with cognitive impairment or otherwise unable to understand the study procedures.	Studies with recruitment based exclusively on one or more specific diagnoses. Excluded populations include, but are not limited to: • Stroke • Parkinson’s (neurodegenerative conditions) • Severe dementia • Long-term care facilities • Housebound • Severe frailty (with protocol for addressing falls or for falls risk assessment in place) • Impaired balance (severe) • Community-dwelling and receiving long-term, intensive nursing care • Visual impairment (severe) • Hospitalized patients • Acute fracture • Confirmed vitamin D deficiency
Exposure(s)	• Experience with fall prevention interventions, or • Exposure to information about different types and/or attributes of falls prevention interventions (e.g., mode, duration, setting, delivery providers, type of intervention); may include information about potential critical outcomes and/or estimates of effect on outcomes from falls prevention interventions, or • No experience or exposure to information about interventions, but authors are soliciting information about preferred intervention attributes Study must relate to types of interventions shown to be effective for at least one critical outcome, from analysis of KQ1. Studies may focus on different attribute(s) of effective interventions, particularly if shown in KQ1 to possibly moderate effects (i.e., specific attributes of interest will be determined based on findings from KQ1)	
Comparison(s)	a) Experience with different type of intervention, or b) Information about a different type of intervention, in terms of its components and/or attributes, or c) No comparison (in studies focusing on attributes within one type of intervention)	
Outcomes	a) Quantitative data about preferences for intervention types or attributes from stated-preference valuation studies (e.g., willingness to pay or accept, preference weights/utility scores, odds ratios, coefficients) b) Quantitative data from non-utility methods, about the relative importance of different intervention attributes (e.g., proportion preferring one type of intervention or attribute, intentions to participate, ranking or ratings of different interventions) c) Qualitative information indicating relative importance between different interventions Outcome groupings (a) to (c) above will be included in a hierarchical manner	
Timing	Follow-up duration: any or none	
Setting	Any	
Study Design and Publication Status	Any cross-sectional or longitudinal quantitative or qualitative study design evaluating preferences between two or more intervention types or attributes of interest, using the methods described below: Methods: a) Contingent valuation or choice experiments (e.g., discrete choice, contingent ranking, or best-worst scaling choice experiment) b) Surveys/questionnaires or studies evaluating decision aids c) Qualitative studies providing information indicating relative importance between benefits and harms Study design groupings (a) to (c) will be included in a hierarchical manner	• Studies only published/available as conference proceedings or other grey literature (e.g., government reports), unless information on study design (e.g., eligibility criteria, participant characteristics, presentation of scenarios) is available (accessible online or via author contact) and sufficient to assess methodological quality. • Commentaries, opinion, editorials, case reports, and reviews
Language	English or French	
Publication date	2000-present	

Abbreviations: *KQ* key question

### Searching the literature

For KQ1 on benefits and harms, we will locate full texts of all studies included in the previous review [29]. Further, a librarian will update this review’s peer-reviewed searches (Additional file 1 contains the search for MEDLINE) from January 1, 2016, in Ovid MEDLINE (1946-), Ovid Embase (1996-), Wiley Cochrane Central Register of Controlled Trials (inception-), and Ageline. The search contains Medical Subject Heading terms and key words combining the concepts of falls/fallers, adults, and randomized controlled trials. Reference lists of all new trials and recent (2018 onwards) systematic reviews will be hand-searched by one reviewer. We will also search the World Health Organization Clinical Trials Search Portal (http://apps.who.int/trialsearch/), which searches multiple trial registries, and ask our clinical experts to provide us with a list of four to five organizational websites to search for conference abstracts and/or reports of research (2018 onwards). Where studies are only reported in conference abstracts or trial registries, first authors will be contacted by email (with two reminders over 1 month) to obtain full study reports and/or additional study or outcome data. If not received, these studies will be excluded with the reason documented.

A search for patient values and preferences (covering both KQs 2 and 3) has been developed combining Medical Subject Heading terms and key words for falls, fractures, and transition to residential care with those for patient preferences, quality of life, various preference–based instrument/methodology terms (e.g., EQ-5D, conjoint analysis), decision making, attitudes, and acceptability (Additional file 1). This search has been peer-reviewed by another librarian using the PRESS 2015 checklist [42]. For this KQ, we will search Ovid MEDLINE (1946-), Ovid PsycInfo (1987-), and CINAHL via EBSCOhost (1937-) databases and hand-search reference lists of included studies and of relevant systematic reviews.

We will export the results of database searches to an EndNote Library (version X7, Clarivate Analytics, Philadelphia, US, 2018) for record-keeping and will remove duplicates. We will document our supplementary search process, for any study not originating from the database searches, and enter these studies into EndNote individually. We will update electronic database searches for all KQs approximately 4 to 5 months prior to publication of the Task Force guideline. Results of new studies will be reported and, if considered to potentially impact conclusions and feasible, the relevant analyses will be re-run.

### Selection of studies

Records retrieved from the database searches will be uploaded to DistillerSR (Evidence Partners Inc., Ottawa, Canada) for screening. For all citations retrieved from the database searches, two reviewers will independently screen all titles and abstracts using broad inclusion criteria. Full texts of any citation from the search considered potentially relevant by either reviewer will be retrieved. One exception is for the study designs in KQ2 on values and preferences that are lowest in our hierarchy (i.e., surveys, qualitative studies), where the full texts will only be reviewed if the other designs offer very low certainty evidence and we proceed to these designs. Two reviewers will independently review all full texts (including the studies from the previous review [29]) against a structured eligibility form, and a consensus process will be used for any full text not included by both reviewers. If necessary, a third reviewer with methods or clinical expertise and/or author contact will be used to arbitrate decisions. The screening and full-text forms will be pilot-tested with a sample of at least 100 abstracts and 20 full texts, respectively, until the agreement is high (> 95%). Screening studies located from reference lists, trial registries, and websites will be conducted by one experienced reviewer, with two reviewers reviewing full texts. Some exclusions are expected to occur after the final groupings/nodes of interventions is conducted (see below), should the study have no comparison between two different groups used for analysis. We will document the flow of records through the selection process, with reasons provided for all full-text exclusions, and present these in a PRISMA flow diagram [43] and appended excluded studies list.

### Data extraction

We will rely on data extraction from the previous review team [29], as able and suitable. Because we are modifying the coding of interventions and adding an outcome of functional status, some data will be required to be extracted anew from the studies included in this review. For this data and for all data from new studies, one reviewer will extract data and another will verify all data for accuracy and completeness. We will adapt the data extraction form and related instructions used by the other review team, as necessary, and provide training for all reviewers involved in extraction. The data extraction form will be piloted with a sample of at least 10 studies, until agreement on all elements is high (> 95%).

Sufficient data will be collected to allow examination of the homogeneity and similarity assumptions for meta-analysis, and for assessment of the risk of bias, as described in the sections below. The main data items include the study characteristics (i.e., year and country of conduct, sample size enrolled, setting of recruitment [hospital vs. other], trial design); intervention(s) components (coded via Table 2), duration (total duration in weeks), dose (number of sessions/hours), assessment and delivery personnel (e.g., primary care provider or team vs. other); description of UC or other control (see Table 2); participant characteristics (sex, age, proportion with previous falls); and outcome tools, ascertainment, and result data (with sample size) at longest follow-up. Although not a focus for the analysis, studies with individuals or populations that may require equity (e.g., Indigenous peoples, newcomers to Canada, low income) [44] considerations by the Task Force will be noted and the applicability of the interventions to these populations will be assessed.

Table 1 contains our outcome definitions. Falls will often be defined as “an unexpected event in which the participant comes to rest on the ground, floor, or lower level” [45] although we will not exclude studies not using this or another definition. Fall-related injuries can be defined in various ways, focusing on symptoms (e.g., limiting one’s normal activities, with or without fracture) and/or resource use (e.g., requiring attendance at the emergency department) [46]. To this end, if a study reports on various related fall-injury outomes, one will be extracted per study using a hierarchy based on assumed severity: falls leading to hospitalization, falls requiring emergency department visit, falls requiring physician visit, or any injurious fall. Of note, the previous review team allowed for data on falls to be included for their outcome of fallers, if the number of fallers was not reported and the number of falls was smaller than the study population. We are keeping these separate because rates of falls may be more sensitive to change than the proportion of fallers [20], and other reviews have found a difference in the effects between falls and number of fallers from falls prevention interventions [18–20]. For the outcomes of injurious falls, fractures, and hip fractures, we will rely on the number of people having one or more event but will include data on the number of events when necessary and assume that a participant would only have one event during follow-up. For the falls outcome, we will use raw data on incidence rates (number of falls per person-year) in each group where available; otherwise, we will calculate incidence rates or use the reported rate ratio (RaR). For the other outcomes, we will extract the crude data on the number of people with the event and the sample size, unless only the risk ratio (RR) or odds ratio (OR) between study groups is reported. If studies report both adjusted and unadjusted ratios, we will use the unadjusted estimate unless the adjustment is for clustering. We will convert RRs to ORs for analysis.

We will record outcome data using an intention-to-treat approach, where possible; if not possible, for instance when only relative effects/ratios between groups are reported instead of raw counts and intention-to-treat not used, we will rely on results from last-observed-carry-forward or, if necessary, per protocol/completer approaches.

When two or more interventions in a three- or four-arm trial are classified as having the same intervention as per our classification (e.g., different intensities of a strength training intervention), we will combine the results from the two interventions [33], to avoid loss of information.

For continuous outcomes measures, we will extract (by arm) the mean baseline and endpoint or change scores, standard deviations (SDs) or other measures of variability, and the number analyzed. If necessary, we will approximate means from medians. If SDs are not given, they will be computed or, if necessary estimated using established imputation methods [33]. When computing SDs for change from baseline values, we will assume a correlation of 0.5, unless other information is present in the study that allows us to compute it more precisely [47]. We will use available software (i.e., Plot Digitizer, http://plotdigitizer.sourceforge.net/) to estimate effects from figures if no numerical values are provided.

We will use an intraclass correlation coefficient (ICC) of 0.01 [48], to adjust findings in cluster-design RCTs that have not done this. We will not adjust studies that randomize by household, considering the likelihood of the clustering effect to be very small [19]. If cross-over trials are included, we will limit the data extraction to the first period of the study, because of the potential for carry-over effects from the nature of fall prevention interventions, and treat the trial as if it used a parallel-group design; the possible unit-of-analysis error introduced is recognized to provide a conservative estimate of the trial effects [33].

For KQs 2 and 3 on patient values and preferences, we will collect data on the population (as per KQ1) as well as exposure to any of the related outcomes and/or to fall prevention interventions. We will extract details about any instrument used, including development and composition of scenarios of health states, choice tasks including definitions of all attributes, or survey questions. Any details provided to participants about the potential benefits and harms of fall prevention interventions will be extracted. Where studies provide results (e.g., health utility values) for more than one type of falls (e.g., people falling once, twice, and more) or fracture outcome (e.g., wrist, tibia, distal femur), we will extract the findings as a range. If including qualitative studies, any relevant section of the results section will be pasted into a Microsoft Excel spreadsheet for further analysis.

We will contact study authors of newly identified studies by email, with 2 reminders over 1 month, if important study data or reporting appear to be missing or are unclear. When there are multiple publications of the same study, we will consider the earliest full publication of the primary outcome data to be the primary data source, while all others will be considered as secondary sources/associated publications. We will extract data from the primary source first, adding in data from the secondary source(s).

### Within-study risk of bias assessments

For KQ1, to align with the previous review conduct [29], we will use the Cochrane Effective Practice and Organisation of Care (EPOC) Group’s risk-of-bias tool [49]. Results by domain for all studies will be reported, although we will also code trials as being at low, moderate, or high risk of bias.

For KQ2, we will use the tool for preference-based studies as per GRADE guidance, which includes questions related to the choice/selection of representative participants; appropriate administration and choice of instrument; analysis and presentation of methods and results; instrument-described health state presentation, of all relevant outcomes and valid with respect to health state; patient understanding; and subgroup analysis to explore heterogeneity [23]. Critical appraisal tools from the Critical Appraisal Skills Programme [50] and the Centre for Evidence-Based Management [51] will be used for qualitative and cross-sectional/survey studies, respectively, in KQs 2 and 3.

For the trials included in the previous review [29], we will rely on the prior assessments by this team. For all other studies, two reviewers will independently assess the studies using the previous team’s reviewer instructions and come to a consensus on the final scores for each question using a third reviewer where necessary. Each risk of bias tool will be piloted with a sample of at least five studies, using multiple rounds until agreement on all elements is high. These assessments will be incorporated into our assessment of the risk of bias across studies when assessing the certainty of the evidence for each outcome (see below).

### Preliminary grouping of intervention components (nodes)

Because there will be the possibility of many different combinations of interventions based on their components, we will form meaningful groups (“nodes” when referring to the NMA) before analysis. After the review team codes all study arms based on their intervention components (Table 2) and other key dimensions (e.g., recruitment setting, delivery personnel), but before any analysis, they will chart the data and consult with the Task Force and clinical experts to create and clarify decision rules for grouping interventions in a meaningful way. The primary consideration will be whether the interventions are considered a single component, multiple components, or multifactorial. Some single-component interventions, differing by single interventions but within the same intervention category in Table 2, may be grouped together (e.g., lighting and flooring). Groupings of different multicomponent and multifactorial interventions may focus on the number of studies to some extent, for example, home hazard assessment and modification combined with exercise may involve different types of exercise if few studies examine each type. Groupings will also focus on factors thought to relate to implementation, such as feasibility, acceptability, access, preferences of patients and providers, and/or modify effects. If requested by the Task Force, we will conduct one or more meta-regressions or stratified analyses using the pair-wise comparisons with UC to see where intervention effects may be modified based on a priori intervention covariates of interest including the inclusion of exercise (in multiple component interventions), dose, intensity, setting, and delivery provider. This would also potentially help prevent heterogeneity in the network meta-analysis. After this process, preliminary networks will be created and the synthesis started. In some cases, the final network configuration may be revised based on the assessment of the NMA, as described below.

### Data synthesis for KQ1 (benefits and harms)

When a meta-analysis is not appropriate, a descriptive summary with accompanying tables and/or figures to present the data will be performed.

NMAs will be considered for all critical outcomes where indirect evidence exists for the outcome and connects to the network. This form of analysis simultaneously evaluates a suite of comparisons. A network of different comparisons is constructed, with nodes representing the different interventions, to consider both direct evidence from comparisons of interest (e.g., intervention B vs. UC) and indirect evidence from other comparisons where one intervention is in common, but not all (e.g., effects from intervention A vs. UC and from intervention A vs. B comparisons will contribute to the estimate of the “network” effect for intervention B vs. UC). For the important but not critical outcome of intervention-related AEs, and for comparisons with UC that are not included in an NMA based on intransitivity or other reasons, pairwise meta-analyses will be conducted where appropriate.

### Pairwise meta-analysis

For pairwise meta-analysis, because of anticipated between-study heterogeneity, we will employ the DerSimonian Laird random-effects model using Stata. For dichotomous outcomes, we will report ORs or RaRs with corresponding 95% CIs. For continuous outcomes, we will report a pooled mean difference using changes scores, when one measurement tool is used. We will use a standardized mean difference when combining two or more outcome scales measuring similar constructs based on clinical input. If suitable, we will transform the results back to the scale most frequently used. If we are not able to use a study’s data in a meta-analysis (e.g., only p values are reported), we will comment on these findings and compare them with the results of the meta-analysis. Where SDs have been imputed or estimated we will perform sensitivity analysis by removing these studies. When event rates are less than 1%, the Peto OR method will be used, unless control groups are of unequal sizes, large magnitude of the effect is observed, or when events become more frequent (5–10%) where the Mantel-Haenszel method without correction factor will be used [52]. The decision to pool studies will not be based on the statistical heterogeneity; the *I*^2^ statistic will be reported but it is recognized that the *I*^2^ is influenced by the number of studies and magnitude and direction of effects [52]. Rather, we will rely on interpretations of the clinical (related to our PICOTS) and methodological differences between studies. When heterogeneity in effects is seen, we will conduct subgroup or sensitivity analysis, using the same variables described in the section on assessment of transitivity in the NMAs. Effect estimates for each outcome will be transformed to risk differences to allow judgment of the clinical importance [53]. For outcomes having statistically significant effects, we will calculate the number needed to treat (NNT) and its 95% CI.

### Network meta-analysis

We will employ random effects NMA and network meta-regressions in the most recent version of Stata available at the time of our analysis, using a frequentist approach that accounts for correlations between effect sizes from multi-arm studies [54]. The measure of treatment effect will be an OR with the exception of the rate of falls where we will report RaRs. The heterogeneity within the same treatment comparison will be measured with the tau-squared which represents the variance of the random effects distribution; this variance will be assumed to be common across the various treatment comparisons although sources of heterogeneity between different comparisons will be explored by network meta-regressions during the assessment of intransitivity.

The assumptions underlying NMA are similar to standard pairwise meta-analysis, but there are additional issues of comparability that need to be considered to ensure the validity of results [55]. Indirect comparisons are not protected by randomization and may be confounded by differences between the trials.

### Assessment of transitivity

Transitivity means that covariates that act as relative treatment effect modifiers are similar across different interventions, or adjusted for using meta-regression, so the effect of all treatments included in the model is generalizable across all included studies. Our exclusion criteria for certain populations expected to require different usual care, and for interventions provided in hospital and home-care settings are thought to prevent substantial intransitivity.

Across studies grouped by comparison, we will investigate the distribution of clinical and methodological covariates that, based on findings from other systematic reviews [19, 20, 29], may be important effect modifiers related to the population or study design―age (< 80 vs. ≥ 80 years), previous fallers (100% vs. > 30 ≤ 100% vs. general population risk of ≤ 30%), recruited at hospitals, countries with the similar healthcare system to Canada (e.g., high-income, Organisation for Economic Co-operation and Development and predominantly universal health care [56]), and study design (i.e., follow-up after randomization [< 12 vs. ≥ 12 months]). We plan to use previous falls rather than increased/high-risk for falls based on various factors, because this is consistently shown to have a strong association with risk for falls (e.g., OR for any fall 2.8 [95% confidence interval 2.4–3.3] and for recurrent fallers 3.5 [95% CI 2.9–4.2]) [57, 58] and there is some evidence to suggest this risk factor alone may modify treatment effects [29].

We will use graphic methods, including weighting edges (lines between nodes) in the network plots based on covariates, to examine similarity between comparisons [38, 55].

Network meta-regressions will also be performed on the NMA to examine the influence of the aforementioned covariates; the change to the heterogeneity (tau value) will be tabulated. If one variable is thought to lead to important statistical heterogeneity, we may verify this with sensitivity analysis and, if necessary, either split the NMA into subgroups using the variables or adjust the NMA for the covariate. Otherwise, the results for relevant comparisons may be rated down for indirectness during the assessment of certainty (see below).

### Assessment of coherence

Incoherence refers to differences between direct and various indirect effect estimates that contribute to the overall “network” estimate for each comparison. We will assess incoherence both locally (per comparison) using the Separate Indirect from Direct Evidence (SIDE, or node-splitting approach [59]) and globally (all treatment effects and all possible inconsistency factors are considered simultaneously) using the design-by-treatment interaction model [54] and comparison of the consistency model to the inconsistency model. These methods provide *p* values, and < 0.01 and < 0.10 may be considered to indicate major and some incoherence [60]. Major global incoherence may result in reconfiguration of the network or not conducting the NMA; otherwise, the degree of incoherence will be considered during the assessment of the certainty of effects as described in that section.

### Presentation of results

We will present all final network plots, with the size of the nodes corresponding to the number of participants randomized to each treatment and the lines/edges weighted by the number of trials evaluating the comparison. The summary ORs or RaRs and 95% CIs for all pairwise comparisons will be presented in a league table (including all direct [where available] and network estimates). To rank the various treatments for each outcome relative to UC, we will use surface under the cumulative ranking curves (SUCRA) and present the SUCRA values in ranking plots; if useful for the working group, we will also create a heat rank plot to display the SUCRA values for all outcomes analyzed. SUCRA values account both for the location and the variance (uncertainty/imprecision) of all relative treatment effects [38]. For each NMA, the overall risk for the UC group will be calculated using the variance-stabilizing Freeman-Tukey double arcsine approach. Network estimates for each node compared with usual care will be transformed to risk differences to allow judgment of the clinical importance [53]. For outcomes having statistically significant effects, we will calculate the number needed to treat (NNT).

### Small study effects

For the NMA outcomes, we will consider using comparison-adjusted funnel plots to assess for small study bias, if clinical input suggests there is rationale for a particular characteristic to be associated with small study effects, and assumptions about the direction of small studies can be made (i.e., treatments need to be ordered in a meaningful way) [38]. Otherwise, we will conduct a funnel-plot grouping all interventions versus usual care, and if bias is evident, we will then assess individual interventions versus UC (if ≥ 10 RCTs) and assess for this bias as usual for pairwise comparisons.

For outcomes where pairwise meta-analysis is used and when 10 or more RCTs are in the comparison, we will analyze for small-study effects both visually using the funnel plot and quantitatively using Egger’s test [61] (continuous outcomes) or Harbord’s test [62] (dichotomous outcomes).

### Data synthesis for KQs 2 and 3 (values and preferences)

This analysis will be guided by a narrative synthesis approach [63]. We will likely rely on textual descriptions and groupings/clusterings to develop a preliminary synthesis of the findings. We will explore relationships between the data by comparing and contrasting study findings while considering study methodology (e.g., timing of outcome measurement), populations (e.g., age, experience with the outcome or intervention type), outcome presentations provided to participants (relevant only for KQ2), comparisons (between outcomes in KQ2 and between differing intervention attributes in KQ3), and analytical approaches. Groupings based on key differences between studies will be created; for example, KQ2 findings from utility and non-utility studies will be separated. Within-study subgroup analyses will be interpreted. We do not anticipate performing meta-analysis, although this may be possible for utility values for some health states/outcomes such as hip fractures if there are two or more studies using the same measurement method in similar populations. If undertaken, we will use a random-effects model. Results for health-state utilities will be separated by utility measurement tool (e.g., EQ-5D, time trade-off) and the main covariates of interest for subgroup analysis will be age, sex, time since fracture (≤ 12 months vs. > 12 months), and fracture history [64].

### Assessing the certainty of the evidence

We will assess the certainty of evidence for all outcomes, for the effects of each intervention grouping versus UC. For outcomes analyzed by pairwise meta-analysis or no meta-analysis, we will follow current GRADE guidance [23, 34, 65–67]. For findings from NMA, we will be guided by the CINeMa approach and use CINeMA software for some assessments, which is based on the GRADE framework, although has conceptual and semantic differences [68]. The assessment covers six domains: within-study bias, across-studies bias (i.e., publication and other reporting biases), indirectness, imprecision, heterogeneity (i.e., variation between studies within a comparison), and incoherence (i.e., variation between direct and indirect sources of evidence across comparisons). Findings during the assessment of transitivity and incoherence of the NMA network will contribute information to support certainty ratings for indirectness and incoherence, respectively, as described further below. Similar to GRADE, judgments for each domain are of no concern, some concern, or major concerns, and for each outcome are of very low, low, moderate, or high. Some of the assessments rely on a percentage contribution matrix (see below). Each outcome starts at high certainty and is rated down for concerns. The six CINeMA domains are interconnected and should be considered jointly rather than in isolation [68]; if two concerns are highly related, we will not rate down twice.

### Percentage contribution matrix

Most studies in a network contribute some indirect information to every estimate of a relative treatment effect. Studies contribute more when their results are precise (e.g., large studies), when they provide direct evidence or when the indirect evidence does not involve many “steps.” The contribution made by each study can be quantified to each relative treatment effect on a 0 to 100% scale. These quantities can be presented in a percentage contribution matrix.

### Within-study risk of bias

CINeMA combines the studies’ percent contributions with the risk of bias judgments to evaluate study limitation for each estimate of a relative treatment effect from a network meta-analysis. More concern about study limitations exists when there is a larger contribution from studies at high or moderate risk of bias. With the percentage contributions, weighted average levels of overall risk of bias are produced. Scores of − 1, 0, and 1 are considered low, moderate, and high risk of bias.

### Across-studies bias

The CINeMA approach provides conditions that would be considered to provide judgments about “suspected” or “undetected” bias. Suspected bias entails (i) failure to include unpublished data, (ii) meta-analysis is based on a small number of positive “early” studies, (iii) the comparison has been funded primarily by industry-funded trials, or (iv) there is existing evidence of reporting bias. A judgment of undetected bias arises from (i) inclusion of unpublished studies with similar findings to those published, (ii) protocols and clinical trial registries are available for many trials and important discrepancies are not found, and (iii) the effects from small studies do not differ from those from large studies [68]. Although our inclusion of gray literature and many studies, as well as the non-pharmacologic topic, would suggest no suspicion of bias, we expect [29] a large portion of the studies to have concerns about selective reporting (e.g., missing outcomes). Outcomes may be rated down if there is evidence of small-study effects or if several studies in the review did not report on the outcome despite inclusion in their protocol and/or when clinical input suggests it should have measured. This approach is very similar to that used for pair-wise meta-analysis.

### Indirectness

Our inclusion and exclusion criteria are fairly rigid and are expected to capture studies of high relevance to the Task Force’s main population, outcomes, and settings of interest. Nevertheless, some comparisons may have some indirectness. Each study included in the review will be coded based on its overall relevance to the main PICOTS (low, moderate, high). Similar to the approach for within-study risk of bias, the findings will then be combined with the percentage contribution of the studies to each comparison to provide a value weighted by each comparison. We will also consider information provided in our assessment of transitivity, when we weighted the edges in the network plots based on covariates in the associated studies to examine similarity between comparisons. If the edges for the comparison are of similar width to those in the majority of comparisons in the network, we will be less concerned about indirectness.

### Imprecision

Imprecision will be assessed in a similar manner as for findings from pair-wise meta-analysis [69]. Because this review is not focusing on the difference in effects between all of the different comparisons, determining a range of equivalence for comparing different interventions (e.g., how much better one needs to be than another) will not be conducted. We will rate down the evidence for imprecision, if, using the network estimate, (i) the effect could be considered clinically important based on the network “point” estimate (e.g., OR ≤ 0.8 for reducing fallers) but the 95% CI crosses the null or (ii) the estimate is likely too small to be important (e.g., OR 0.95) but the 95% CI includes values indicating the possibility of an important effect in either direction. Rating down by two levels may occur if the effect appears to be of little to no difference but the 95% CI is very wide, indicating possible benefit and harm (e.g., spanning ORs of both < 0.75 and > 1.25) [69].

### Heterogeneity

The concordance between assessments based on confidence intervals, which do not capture heterogeneity, and prediction intervals, showing where the true effect of a new study similar to the existing studies is expected to lie, can be used to assess the importance of heterogeneity. The effect of the heterogeneity on the conclusions will be considered (see imprecision for general rules on effect sizes), and if the predictive intervals do not add any concern over that already assessed for imprecision, we will not rate down for this domain. Predictive intervals derived from meta-analyses with very few studies can be unreliable and this will be taken into account.

### Incoherence

We will use results from our local (per comparison; using SIDE, or node-splitting approach [59]) and global [54] assessments of incoherence. Both methods provide *p* values, and we will consider < 0.01 and < 0.10 to indicate major and some incoherence [60]. Comparisons that have > 90% direct evidence will not be rated down. For comparisons that have only indirect evidence (i.e., local coherence not relevant), we will rate down due to incoherence one or two levels depending on whether the *p* value of the design by treatment interaction model was between 0.01 and 0.10 or less than 0.01, respectively. If there is > 0% and < 90% direct evidence, we will base the decision on the more relevant method (e.g., high reliance on node splitting when more direct evidence). We will also consider the 95% CIs from the direct and indirect evidence for each comparison; if both are showing the same direction of effect, but differing magnitudes of beneficial effects, we will have less concern.

Input from the Task Force will be used when the review team conducts the certainty assessments for each outcome, for example, when appraising the applicability/indirectness of the studies in terms of the population of interest to their recommendation.

## Discussion

The review will be published in an open-access journal and reported using standard checklists for systematic reviews and network meta-analysis [70, 71]. The results section of the review will include a description of the flow of literature and characteristics of all studies, results of all analyses, including planned subgroup and sensitivity analyses as well as the assessment of the NMAs, and summary of finding tables incorporating assessment of our confidence in the estimates of effect. In the discussion, we will summarize the main findings and their implications, compare our findings to others, and discuss the limitations of the review and the available literature. The results will be used by the Task Force for developing recommendations about fall prevention in community-dwelling older adults. It will also serve as a comprehensive review for clinicians and other decision makers on the effects of interventions and relevant patient preferences.

### Protocol amendments

We will report on any deviations from the protocol within the final manuscript.

## Supplementary Information

**Additional file 1.**

## Data Availability

Not applicable.

## Abbreviations

ADLs: Activities of daily living
CI: Confidence interval
EPOC: Cochrane Effective Practice and Organisation of Care
GRADE: Grading of Recommendations Assessment, Development and Evaluation
ICC: Intraclass correlation coefficient
KQ: Key question
NMA: Network meta-analysis
OR: Odds ratio
PHAC: Public Health Agency of Canada
PICOTS: Population, interventions, comparators, outcomes, timing, setting
RaR: Rate ratio
RCT: Randomized controlled trial
RR: Relative risk
SD: Standard deviation
SIDE: Separate indirect from direct evidence
SUCRA: Surface under the cumulative ranking
UC: Usual care
