# Supplementary material for: Fall prevention interventions for older community-dwelling adults: systematic reviews on benefits, harms, and patient values and preferences
Source: Syst Rev. 2021 Jan 9;10:18. doi: 10.1186/s13643-020-01572-7 (PMC7797084; doi:10.1186/s13643-020-01572-7)
Supplement: Supplementary file 1 — Additional file 1. [file 13643_2020_1572_MOESM1_ESM.docx]

**Additional file 1: Medline search strategies**

**Key question 1**

| 1 | Accidental Falls/ |
| --- | --- |
| 2 | (fall or falls or fallen or faller? or falling or fell).ti,ab. |
| 3 | (fall-related or falls-related or near-fall?).ti,ab. |
| 4 | 1 or 2 or 3 |
| 5 | Middle aged/ |
| 6 | Aged/ |
| 7 | aged.tw,kf. |
| 8 | (elderly or geriatric* or gerontolog* or old-age? or senior?).tw,kf. |
| 9 | (older adj2 (adult* or female? or male? or man or men or patient? or person? or people? or population? or woman or women)).tw,kf. |
| 10 | or/5-9 |
| 11 | randomized controlled trial.pt. |
| 12 | randomi#ed.ti,ab. |
| 13 | randomly.ti,ab. |
| 14 | placebo.mp. |
| 15 | or/11-14 |
| 16 | 4 and 10 and 15 |
| 17 | exp Animals/ not (humans/ and exp animals/) |
| 18 | 16 not 17 |
| 19 | limit 18 to (yr="2016 -Current" and (english or french)) |

**Key questions 2 and 3**

| 1 | Accidental Falls/ |
| --- | --- |
| 2 | (fall or falls or fallen or faller? or falling or fell).ti,ab. |
| 3 | (fall-related or falls-related or near-fall?).ti,ab. |
| 4 | 1 or 2 or 3 |
| 5 | Fractures, Bone/ |
| 6 | Fractures, Spontaneous/ |
| 7 | Hip Fractures/ |
| 8 | Osteoporotic Fractures/ |
| 9 | ((bone? or hip?) adj3 (break* or broke? or fractur*)).tw,kf. |
| 10 | ((spontaneous* or pathologic*) adj2 fractur*).tw,kf. |
| 11 | or/5-10 |
| 12 | Institutionalization/ |
| 13 | exp Residential Facilities/ |
| 14 | exp Nursing Homes/ |
| 15 | institutionaliz*.tw,kf. |
| 16 | assisted living.tw,kf. |
| 17 | nursing home*.tw,kf. |
| 18 | (residential adj1 (home* or facilit* or care)).tw,kf. |
| 19 | (home adj3 (aged or elder* or geriatric)).tw,kf. |
| 20 | or/12-19 |
| 21 | Patient Transfer/ |
| 22 | Transitional Care/ |
| 23 | ((new* or recent*) adj admit*).tw,kf. |
| 24 | transition*.tw,kf. |
| 25 | transfer*.tw,kf. |
| 26 | (change or changing).tw,kf. |
| 27 | await*.tw,kf. |
| 28 | relocat*.tw,kf. |
| 29 | (move? or moving).tw,kf. |
| 30 | or/21-29 |
| 31 | 20 and 30 |
| 32 | Patient Acceptance of Health Care/ |
| 33 | Patient Participation/ |
| 34 | Attitude to Health/ |
| 35 | ((attitude* or accept*) adj3 (client* or person* or people* or individual* or female* or male* or men or patient* or wom#n*)).tw,kf. |
| 36 | or/32-35 |
| 37 | Informed Consent/ |
| 38 | Choice Behavior/ |
| 39 | Decision Making/ |
| 40 | Patient Preference/ |
| 41 | *"Quality of Life"/ |
| 42 | (15D* and (HRQoL or QoL or "quality of life")).mp. |
| 43 | ((analys#s or valuation? or value? or valuing) adj3 (conjoint or contingent)).tw,kf. |
| 44 | (choice? adj2 (behavio?r* or discrete or experiment*)).tw,kf. |
| 45 | ((choice? or choos* or consent* or decision*) adj1 informed).tw,kf. |
| 46 | (EQ?5D* or EQ5D* or EuroQoL 5D or EuroQoL5D).tw,kf. |
| 47 | disabil* weight*.tw,kf. |
| 48 | (health adj2 utilit*).tw,kf. |
| 49 | HUI?.tw,kf. |
| 50 | (multi?attribute or multi?criteria).tw,kf. |
| 51 | (pay adj2 willing*).tw,kf. |
| 52 | preference*.tw,kf. |
| 53 | prospect theor*.tw,kf. |
| 54 | (QoL or quality of life).ti. |
| 55 | (SF?12 or SF?36 or SF?6D or SF12 or SF36 or SF6D).mp. |
| 56 | standard gamble*.tw,kf. |
| 57 | (trade off? or tradeoff? or trade-off?).tw,kf. |
| 58 | (willing* adj2 pay*).tw,kf. |
| 59 | ((choice? or choos* or chose? or decid* or decis* or preference*) adj3 (client* or female* or male* or men or patient* or wom#n*)).tw,kf. |
| 60 | best-worst.tw,kf. |
| 61 | or/37-60 |
| 62 | ((4 or 11 or 31) and 61) or (4 and 36)) |
| 63 | limit 62 to (yr="2000 -Current" and (english or french)) |
